# New opto-electro-mechanical sensor for two-dimensions dosimetry based on radiochromic films

**DOI:** 10.1038/s41598-023-43387-1

**Published:** 2023-10-05

**Authors:** S. Mena, N. Karkour, V. Alaphilippe, J. P. Botero, M. Jiménez, D. Linget, L. Gibelin, V. Le Ven, A. Marquet, S. Mellouh, E. Josson, W. Benassou, X. Muñoz-Berbel, G. Guirado, C. Guardiola

**Affiliations:** 1 Departament de Química, Universitat Autònoma de Barcelona, 08193 Bellaterra, Barcelona Spain; 2 Instituto de Microelectrónica de Barcelona, (IMB-CNM, CSIC), 08193 Bellaterra, Spain; 3 Université Paris‒Saclay, CNRS/IN2P3, IJCLab, 91405 Orsay, France; 4 Universidad de los Andes, Carrera 1 No. 18ª-10, Bogotá, Colombia; 5 ESME (École Supérieure Mécanique et Electronique) Sudria, Paris, France; 6 Faculté de Chimie, Université Paris-Saclay, Orsay, France; 7 CIBER de Bioingeniería, Biomateriales y Nanomedicina, Instituto de Salud Carlos III, Madrid, Spain

**Keywords:** Radiotherapy, Applied physics

## Abstract

This work presents the validation of a new Opto‒Electro-Mechanical (MOEM) system consisting of a matrix of photodetectors for two-dimensional dosimetry evaluation with radiochromic films. The proposed system is based on a 5 × 10 matrix of photodetectors controlled by both in-house electronic circuit and graphical user interface, which enables optical measurements directly. We present the first tests performed in an X-ray machine and ^137^Cs source with that array by using Gafchromic EBT3 films. We obtained similar results than with a standard method (e.g. flat-bed scanner). Results were compared with Monte Carlo simulations and very good agreement was found. Results show the feasibility of using this system for dose evaluations. To the best of our knowledge, this is the first MOEM sensor for radiotherapy. Further developments are ongoing to create an advanced 16 × 16 LDRs system covering 1.6 cm × 1.6 cm with a 1 mm of spatial resolution. We point to develop a portable dosimetry tool delivering dose maps in real time to improve the clinical application of radiochromic films.

## Introduction

Irradiation in medicine, chemistry or security is the process by which an object is exposed to ionizing radiation. The effects of ionizing radiations depend on the type of radiations and the dose delivered, which is the energy deposited locally by unit of mass. A precise determination of the dose locally delivered to an object is of interest in multiple fields. For example, in radioprotection, the continuous monitoring of radiation doses is necessary to guarantee the safety and security of the people, and to adapt the protection tools to it. In the case of radiotherapy, the measurement of the radiation dose has a twofold function. On the one hand, it permits the validation of the machine’s effective operation for dose delivery (i.e. beam output verification) within the specific conditions recommended by international protocols. On the other hand, it ensures that the prescribed dose is properly delivered to the patient at the targeted location. Regarding to the latter, radiochromic films (RCFs) are currently the gold‒standard passive detectors for the measurement of dose distribution in radiology and radiotherapy^1–3^. These RCFs are used for beam position alignment, measuring beam slice-width or the surface skin-dose in interventional procedures, among others^4,5^. Likewise, they are widely employed for quality assurance measurements in photon, electron, and proton therapy^6–9^ thanks to their sub-millimetre spatial resolution, low energy dependence, quasi water‒equivalence, sensitivity to a wide dose range (from few mGy to hundreds of kGy) and overall easy use^4,10^.

Under exposure to ionizing radiations, a polymerization reaction occurs within the active layer (mainly diacetylene) of the RCF, which generates colored polymer chains and thus a change in the film color. This color change depends on the radiation dose absorbed by the film and correlates well to the delivered dose without additional chemical post-processing (self-developing). The net optical density (OD) of a film at a specific dose represents the difference of OD after and before of being irradiated. Currently, the standard quantification method of these OD changes is based on the use of image scanning instruments based on densitometers, e.g., flatbed scanner, which requires a minimum of 24 h of self-development of the film before scanning^4,10,11^. Hence, there is a delay between irradiation and scanning, and the subsequent evaluation of the dose delivered.

To reduce decision making, the dose delivered is generally calculated with analytic models implemented in the radiotherapy software of the treatment planning systems. In this context, the time-delay of the radiochromic films makes these models an unfeasible tool for real-time daily dose evaluation and, when the clinic workflow requires a dose verification in real time, films information is complemented with active radiation sensors including semiconductor and scintillation detectors, or gas chambers^10^. Therefore, there is still room for developing a device that allows measuring in real-time and in situ the dose.

In response to these issues, we have designed and created a new two-dimensional sensor that consists of a matrix of micro-opto-electro-mechanical (MOEM) elements^12,13^ by using potential values of the system output as dose transducer. It consists of an optical sensor with light emitting diodes (LEDs) and 5 × 10 light-dependent resistors (LDRs) integrated in a fully tailored mechanical and electrical system including an in-house data analysis software and a graphical user-interface. A patent has been filled in March 2021^12^. We present here a validation test performed with this prototype. Tests were performed irradiating Gafchromic EBT3 films with X-rays and a ^137^Cs source. Results show the feasibility of using this system for further dose evaluation.

To the best of our knowledge, this is the first matrix of MOEMs as an active sensing system for fast radiochromic film analysis.

## Methods

### Radiochromic films

Dose evaluations were characterized with Gafchromic™ EBT3 films (Gafchromic, International Specialty Products, Wayne, New Jersey, USA) that is mainly used for external beam therapy, radiosurgery, and brachytherapy QA^14^. They are crystalline polyacetylene based radiochromic films that contain a yellow marker dye^3,5,10,11^. The optimal dose range of these films is between 0.2 and 10 Gy, with uniformities better than ± 3% in dose, according to the supplier. These films are coated on special polyester to avoid Newton’s rings patterns and show a spatial resolution up to 25 µm^14^.

### Scanner system

Absolute dose evaluations of the irradiated films were obtained following well stablished protocols for Gafchromic EBT3 films^15^. The response of irradiated films was evaluated in terms of their OD changes before and after irradiation. To quantify it, we used a flat‒bed colour scanner in transmission mode. These scanners measure the red, green, and blue colours of light transmitted by the films after irradiating with broad band fluoroscopic visible light sources. OD changes were assessed as a logarithm of the inverse transmission (Eq. 1):

1 $$netOD={OD}_{after}-{OD}_{before}=-{log}_{10} \left(\frac{I}{{I}_{0}}\right)$$

where I_o_ and I represent the intensity of the light passing through the unexposed (control) and irradiated films respectively. Previous calibration curve to convert changes of OD into absolute dose is mandatory. It was performed by correlating the dose deposited inside the Gafchromic EBT3 films with a reference dosimetry system (see below in “X-ray irradiation” section). Gafchromic EBT3 films were calibrated in a configuration where they were placed perpendicularly to the beam direction. Similar region of interest (ROI) was defined over the irradiated samples (4 cm × 4 cm) around the beam axis, and mean pixel values of the transmission scan were recorded. NetOD was calculated as Eq. (1) with these values. Reading evaluations were calculated using an in‒house code regarding the calibration curves. Dose measurement uncertainties were below 5%.

Films handling was performed following the recommendations provided by Task Group 55 of the AAPM^15^. A flat-bed RGB scanner (Epson Perfection V700 photo scanner) was used for the readout at 1200 dpi resolution. Images were saved in tagged image file format (tiff) and later analysed with ImageJ^16^. We followed the methodology described by Devic et al.^17^ and used the red channel, which is recommended for dose evaluation up to 8 Gy.

### Sensor system (“*dosiMOEMs*”)

The sensing system was developed by the Electronics Department in the *Laboratoire de Physique des 2 Infinis Irène Joliot-Curie* (IJCLab, CNRS). It consists of an opto-electron-mechanical (MOEM) system with two modules: the first one is the light source composed by high precision and low noise variable 3 × 3 light emitting diodes (green LEDs) controlled independently, and the second one is the sensor containing a matrix of 5 × 10 resolution light dependent resistors (LDRs).

On the one hand, an in-house Data Acquisition (DAQ) controls the 3 × 3 LEDs through a precision ADC and uses a serial port to activate and disactivate them through a micro analog switch, which can be adjusted with the software below. The final prototype used a very low noise amplifier plus low pass filter with PDV8101 LDRs. The light emitted is a common analog value that varies between 0 and 3 V with 256 steps with a low-pass-filter and a high precision amplifier driver, which delivers precise voltage values to enable constant light emission.

On the other hand, the photodetector is controlled by the DAQ system that digitizes and scans all the LDRs, one by one several times, to generate an average LDR outcome-value through an ADG731 multiplexer system. The DAQ system is based on an ESP32 microcontroller whose linearity was qualified using high precision resistors and LDRs. The LDR readout selection is done using micro analog switches connected between the LDR and the 32-input multiplexer. The output of the multiplexer is connected to a variable gain amplifier to adjust the gain to cover all the 12 bits ADC range (from 0 to 4096). To maximize the system efficiency, the gain of the LDR readout amplifier was fixed by using the range of the digitized values of a set of irradiated films in X-ray from 0 to 10 Gy. The overall intrinsic resolution of the system (LED + LDR + ADC) is < 0.2%.

Both modules are covered by a black cage to avoid background noise and the system is powered by a battery to remove the noise from USB or external power supplies.

The samples, i.e. the irradiated films, are located between both modules individually. When the LED module is activated, the corresponding green light goes through the sample. The transmitted light reaches then the detector module and provokes a resistance decay of the LDR, which is inversely proportional to the amount of light received. In order to read the on‒the‒spot resistance, the signal is converted to a potential value. We performed a study testing 20 commercial LDRs to obtain the optimal one. Both, low and high range resistance LDR ranges were tested. The qualification was made through the three LED colours, being the green one the most suitable in terms of resolution for the full range of the ADC.

The communication system is based on an in‒house python‒based software, using a graphic user interface developed with the Tkinter package. It allows us to control the hardware, e.g., control the duration of the acquisition, the LED light intensity, the LDR and LED distance (by means of a micromanipulator). The code generates an automatic qualification (ADC, LDR, resistances, statistical, etc.) that generate the dose values for each LDR. All the LDR multiplexed uses 1 ADC input, which reads each LDR several times (programmable value) to reduce the glitch noise and calculates the average value to be stored in the memory. Once the 50 channels are read out, the ESP32 sends the data to a graphic interface (< 1 min). It also saves the data for further post processing and analysis.

Following that method, measurements with the green lights were performed systematically. As a first approach to compare the performance of our system with that obtained with the standard method, we performed the measurements 48 h after the irradiation, as it is recommended in the standard protocols^15^. Note that only 20 LDRs (the top ones) were used since the irradiated film size (3 cm × 3 cm) was covered with those LDRs. Outcomes are shown in the  “Results”  section.

### X-ray irradiations

Irradiations were performed using a 90 kV microfocus X-ray source L9421-02 (Hamamatsu) available in the IMB-CNM (CSIC). It can operate from 20 to 90 kV, from 10 to 200 µA, has a focal spot size of 7 µm with 39^0^ aperture and a focus to object distance of 9.5 mm. The target material is tungsten, and the system has a 150 µm Beryllium windows. The irradiations were performed at 60 kV, 120 mA and at 140 mm of distance (source-sensor-distance (SDD) of 79.5 mm). Due to the complexity of X-ray spectrometry, some semiempirical models for generating the X-ray spectra from tungsten targets have been proposed^18,19^. The energy distribution generated by this machine was obtained by using the Xcomp5r software, a DOS program in BASIC code^20^ that implements these models. We used 60 kV with a 1.3 mm of Aluminium as filter along all the tests.

The corresponding calibration of the X-ray machine was determined with a plane parallel chamber (Raysafe, X2 R/F sensor), of an active sensor area of 9 mm × 4 mm, previously calibrated in terms of air kerma free-in-air (Kerma_air_), which is typically used for QA measurements on X-ray machines. The uncertainty range found for 60 kV voltage was between 5 and 10%. Therefore, a conservative dose uncertainty of 10% will be considered in all the cases below. The calibration curve is given as a function of the SSD:

2 $$Dose \; (mGy)=138251\cdot {(SSD-{D}^{\prime})}^{-2.003}$$

where D′ is an offset that was obtained calculating the dose versus time for two SSD distances. It is important to note that, this X-ray machine is not optimized for irradiating large surfaces. Thus, the irradiated area may lack homogeneity. We quantified the inhomogeneity of the irradiation field by using an in-house C++ code. Results are displayed below.

### ^137^Cesium source irradiations

Additional irradiations were carried out at the Technical Protection Unit radiological (UTPR) of the Universitat Autònoma de Barcelona. The irradiator was a ^137^Cesium source, IBL 437C, type H (SCHERING CIS Bio International, No. 701, 93/42/CEE). The equipment was calibrated according to DS/53-00-60-10 CIS bio international procedure by using Alanine dosimeters from the Gammaservice laboratory (calibrated by the Physical Technical bundesanstalt in Deuschland and the National Physical laboratory in England). Alanine dosimeters were placed in the centre of the canister filled with water. The dose rate was 4.05 Gy min^−1^ with a dose uncertainty of 3.5% according to the calibration. In this work Gafchromic EBT3 films were also placed into the canister, but irradiations were performed in the air.

### Monte Carlo simulation

The simulation code PENELOPE/penEasy-IR has been used^21^. It is an adaptation of the PENELOPE/penEasy code developed to obtain dosimetry personnel in interventional radiology conditions. PenEasy is a main program structured for PENELOPE that facilitates the inputs/outputs that must be generated by the user.

The dose in PENELOPE/penEasy-IR was calculated from the fluence provided by the *Tally Photon Fluence Point* at the point of interest. The X-ray machine above was simulated regarding the manufacturer specifications. Likewise, the geometrical settings and the gafchromic films were simulated. The simulated parameters were: 25° anode angle, 19.5° beam opening angle, RX output spectrum (60 kV, 0.15 mm beryllium window). The cut-off values of the secondary electrons and photons (WCC, WCR respectively) were the default. The parameters that determine the mean free path between two elastic events (C1) and the average maximum fraction of energy loss (C2) were also the default ones (between 0 and 0.2).

## Results

A set of Gafchromic EBT3 films were irradiated, on the one hand, in X-ray irradiators from 0.2 to 8 Gy and, in the other hand, in and ^137^Cs irradiator from 0.2 to 4 Gy. Afterwards, films were kept in the dark for better conservation. Films were analysed after 48h using both the conventional optical densitometer (“Scanner system” section) and our system (“Sensor system (“*dosiMOEMs*”)” section) to evaluate the OD changes in the irradiated films and the corresponding performance of *dosiMOEMs* versus a standard method.

### X-ray irradiations

#### Analysis with scanner

**Figure 1:**
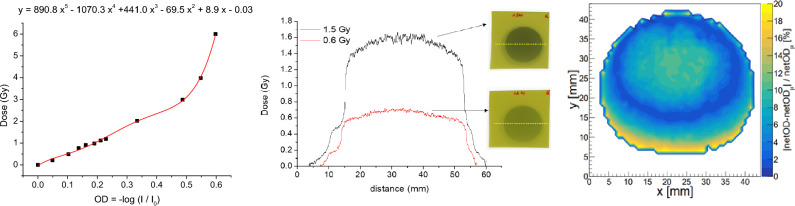
*Left* Calibration curve obtained by the scanner method. *Medium* dose profiles of the two films irradiated with 0.6 and 1.5 Gy. The insets show both gafchromic films irradiated. The yellow dash lines represent the lateral sections where the dose profiles were extracted from. *Right* Measured dose distributions that show the displacement of the beam intensity in terms of the percentage of OD dispersion for a gafchromic EBT3 film irradiated at 4 Gy.

In Fig. 1, left, it is depicted the calibration curve by plotting the netOD versus the dose (Gy). It was extracted by fitting the data with a fifth-grade polynomic function after the post-processing. Then, it is possible to evaluate an unknown dose through an in‒house code including such calibration curve. Using the program ImageJ^16^, the scanning image of the irradiated Gafchromic EBT3 film is transformed into an OD profile versus the analysed distance. We converted this OD profile into dose values through the in-house C++ code. As a figure of merit, it is shown in the Fig. 1, medium, the dose profiles of two films irradiated with values inside of the calibration curve, but at different doses. Due to the X-ray machine nature, the irradiation fields were not completely homogeneous, and the curve shapes in Fig. 1, medium, were not completely straight. The average dose was (0.63 ± 0.07) Gy and (1.5 ± 0.2) Gy, for the nominal doses of (0.60 ± 0.06) Gy and (1.5 ± 0.2) Gy, respectively. Note that while the netOD random errors were calculated by the standard deviation of the mean pixel value, the final overall uncertainty was limited by the systematic error of the X-ray machine above, i.e. 10% of the dose (“X-ray irradiation” section).

**Figure 2:**
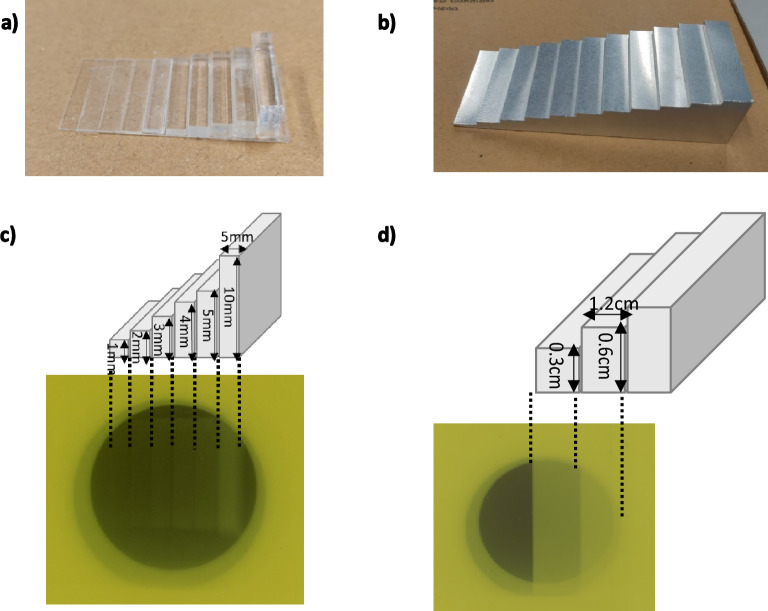
(**a**) PMMA and (**b**) Aluminum stairs. (**c**,**d**) sketches and dimensions of both staircases and the corresponding images of both gafchromic films irradiated.

Inhomogeneity was then quantified using another in-house C++ code. Fig. 1, right, shows the percentage of the OD dispersion along one representative irradiated Gafchromic EBT3 film (4Gy), obtaining a maximum OD difference (Equation 3) of 20%. It gives us a comprehensive assessment of the field distribution that will be used afterwards.

3 $$\%OD\; dispersion=\frac{\left|netOD- {netOD}_{\mu }\right|}{ {netOD}_{\mu }}$$

where %OD dispersion is the percentage of optical density changed. NetOD is the total optical density of the film and netOD_μ_ is the optical density which changed in the film. To assess the resolution accuracy of both systems, firstly, we created two patterns in the format of stairs to obtain heterogeneous dose distributions: (i) a PMMA mould (Fig. 2a), which was home-made adding simple pieces of PMMA of 5 mm × 25 mm but different height (from 0.18 to 10 mm) and an Aluminium (Al) piece (Fig. 2b). They have an increasing radiation absorption capacity that create a decreasing dose gradient (*dose-steps*) in the films. The six PMMA steps which were used for creating the dose-step film have 5 mm width, a constant pitch (5 mm), and 1 mm height [except for the last one of 10 mm height (Fig. 2c)]. The Al stairs steps have 1.2 cm width with an increasing 3 mm height stepwise. It was only possible to distinguish three zones in the irradiated EBT3 film (Fig. 2d). A piece of film was placed below each stair and irradiated at nominal dose of 3Gy.

**Figure 3:**
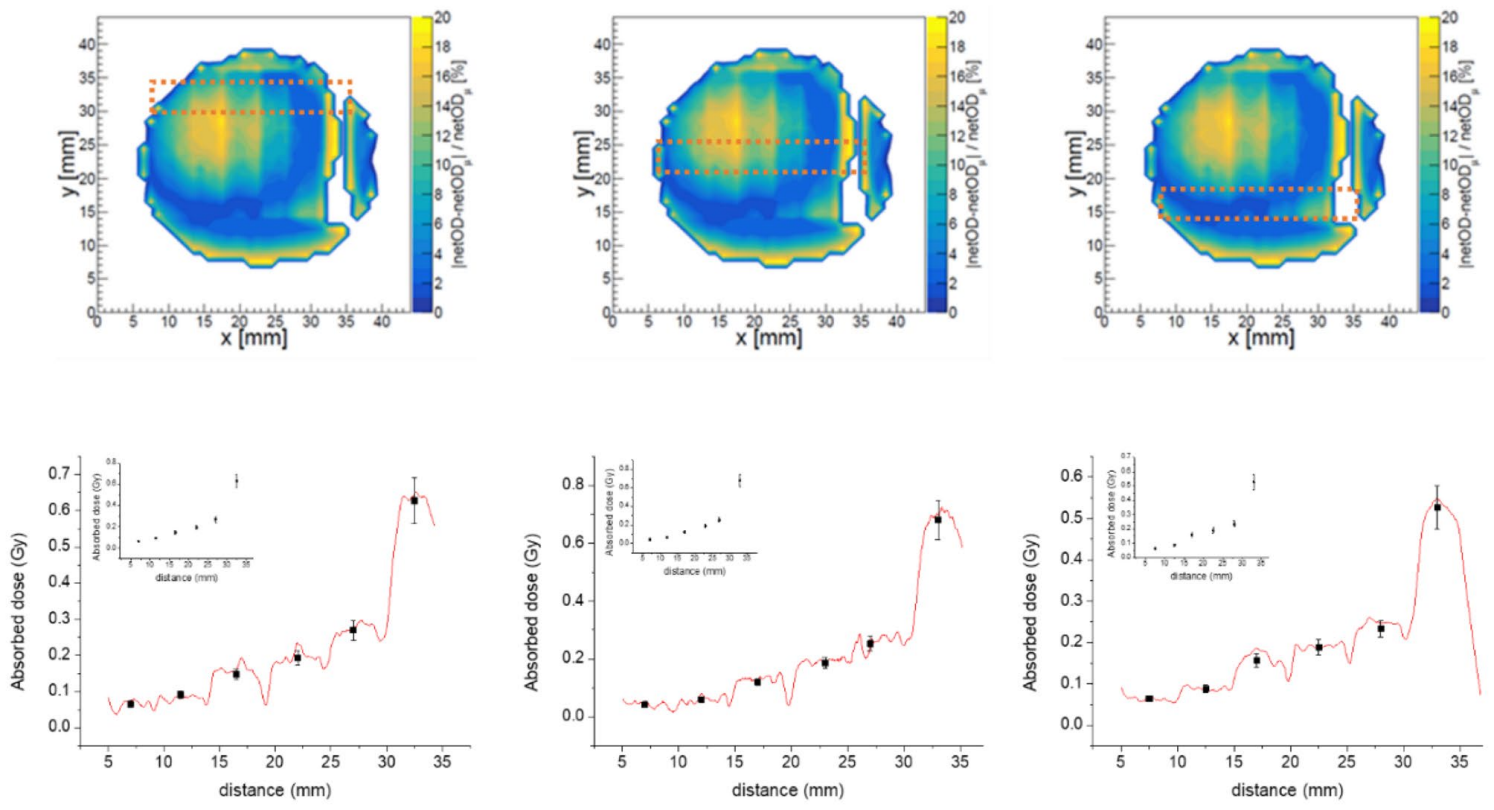
*Top* Maps of the percentages of the OD dispersion for the Gafchromic EBT3 film irradiated at nominal 3 Gy with the PMMA staircase for three zones (top, middle, bottom). Orange lines indicate the zone selected for the studied profile. *Bottom* absorbed doses in each PMMA step once the *background* is subtracted. The inset shows the average values in the middle of the steps (black dots) corresponding to each PMMA step.

Although the PMMA steps were distinguishable in all cases (Fig. 3, up), we observed high dispersions depending on the irradiated zone, such as it was shown in Fig. 1, right, due to the intrinsic heterogeneity of the X-ray machine. For this reason, the irradiated film was divided in three zones (top, middle, and bottom) that were analysed independently. A further analysis requires the subtraction of the OD values from those obtained with an irradiated film at the same dose (without the PMMA stairs). Figure 3, bottom, shows the doses absorbed by each PMMA step in the three zones above, obtaining consistent values into the error bars.

Table 1 (entries 1–6) summarizes the results. Similar values into the error bars were obtained for the three positions along the film. Nevertheless, it is observed that the absorbed dose below the 1 mm-height PMMA is like the obtained for the 2 mm-height step. It may be due to the inhomogeneity of the films (with dose variations up to 20%) that, joined to the manual alignment, might generate non-negligible OD variations because of an inaccurate subtraction of the background. These values (*middle position*) were compared with those obtained with *dosiMOEMs* in Table 2.

**Table 1:** The nominal dose delivered is 3 Gy.

Entries	PMMA staircase			
	Measured dose (Gy) in the EBT3 film			
	PMMA step height (mm)	Upper position	Middle position	Bottom position
1	1	2.9 ± 0.3	2.9 ± 0.3	2.9 ± 0.3
2	2	2.9 ± 0.3	2.9 ± 0.3	2.9 ± 0.3
3	3	2.9 ± 0.3	2.9 ± 0.3	2.8 ± 0.3
4	4	2.8 ± 0.3	2.8 ± 0.3	2.8 ± 0.3
5	5	2.7 ± 0.3	2.8 ± 0.3	2.8 ± 0.3
6	10	2.37 ± 0.24	2.32 ± 0.23	2.47 ± 0.25

	Al staircase			
	Measured dose (Gy) in the EBT3 film		Monte Carlo simulation	
	Al step height (cm)		Experimental dose ratio	Simulated dose ratio
7	0	2.9 ± 0.3		
8	0.3	0.98 ± 0.10	2.96 ± 0.61	3.08 ± 0.06
9	0.6	0.49 ± 0.05	5.92 ± 1.22	5.77 ± 0.13

The consecutive measured doses in the EBT3 film, which is under the PMMA & Al stairs, is progressively reduced accordingly to the absorbed dose in each PMMA (or Al) stairs-step. For the experimental and simulation dose ratio values, it was considered as reference the dose without Al staircase, entry 7.

**Table 2:** The nominal dose delivered is 3 Gy.

PMMA step height (mm)	Measured dose (Gy) in the EBT3 film			Monte Carlo simulation	
	Scanner	dosiMOEMs (position #1)	dosiMOEMs (position #2)	Experimental dose ratio	Simulated dose ratio
1	2.9 ± 0.3	–	2.8 ± 0.3	1.07 ± 0.23	1.04 ± 0.02
2	2.9 ± 0.3	2.7 ± 0.3	2.7 ± 0.3	1.11 ± 0.23	1.09 ± 0.02
3	2.9 ± 0.3	2.6 ± 0.3	2.6 ± 0.3	1.15 ± 0.25	1.13 ± 0.02
4	2.8 ± 0.3	2.5 ± 0.3	2.6 ± 0.3	1.15 ± 0.25	1.17 ± 0.02
5	2.8 ± 0.3	2.5 ± 0.3	2.4 ± 0.3	1.20 ± 0.26	1.21 ± 0.02
10	2.32 ± 0.23	2.1 ± 0.2	–	1.43 ± 0.28	1.42 ± 0.02

The consecutive measured doses in the EBT3 film, which is under the PMMA stairs, is progressively reduced accordingly to the absorbed dose in each PMMA stairs-step. For the experimental dose ratio values were considered the measured dose (Gy) obtained with *dosiMOEMs*. Note that for experimental and simulation dose ratio values, it was considered as reference the dose without PMMA staircase.

On the other hand, Table 1 (entries 7–9) shows the results as another set of Gafchromic EBT3 films were irradiated at 3 Gy with the Al staircase. As it is expected, the X-ray absorption of the Al staircase was higher than that with PMMA. The dose measured was (2.9 ± 0.3) Gy without stairstep, (0.9 ± 0.3) Gy, and (0.49 ± 0.05) Gy by the steps of 0.3 and 0.6 cm, respectively. In this case, was performed a Monte Carlo simulation and the experimental dose ratios (considering as reference the dose without Al staircase, i.e. 0 cm) and the simulated ones are in good agreement into the error bars.

#### Analysis with dosiMOEMs

**Figure 4:**
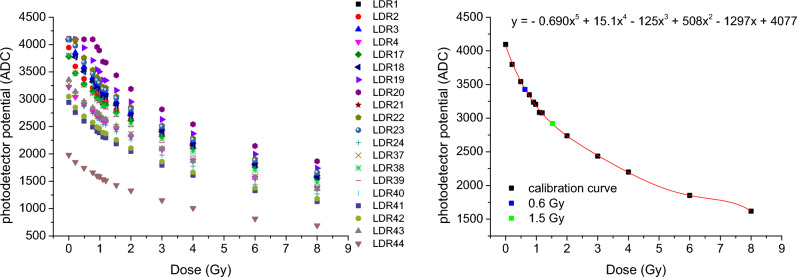
*Left* calibration curve obtained for each LDR. *Right* normalized calibration curve and absorbed dose values obtained for two films irradiated at 0.6 and 1.5 Gy.

Figure 4, left, shows the different calibration curves obtained for the 20 LDRs used *dosiMOEMs* (see “Sensor system (“*dosiMOEMs*”)” section), which were extracted by fitting the data with a fifth-grade polynomic function. Although the 20 LDRs belong to the same type, it was necessary to create a matrix of conversion factors between the fitted functions of each LDR and one of reference (e.g., LDR#1) to correct any manufacturing differences. The final calibration curve after correction, valid for all LDRs, is illustrated in Fig. 4, right, (dark dots). Then, it was possible to characterize unknown doses by converting in-situ the potential values (ADC *in dosiMOEMs*) into dose without post-processing. For example, two films (blue and green dots in Fig. 4, right) were irradiated using different irradiation times than those used for the calibration. Doses of (0.6 ± 0.1) Gy and (1.5 ± 0.2) Gy were obtained, which was consistent with the corresponding nominal dose calculated with the X-ray machine calibration according to the irradiation time.

**Figure 5:**
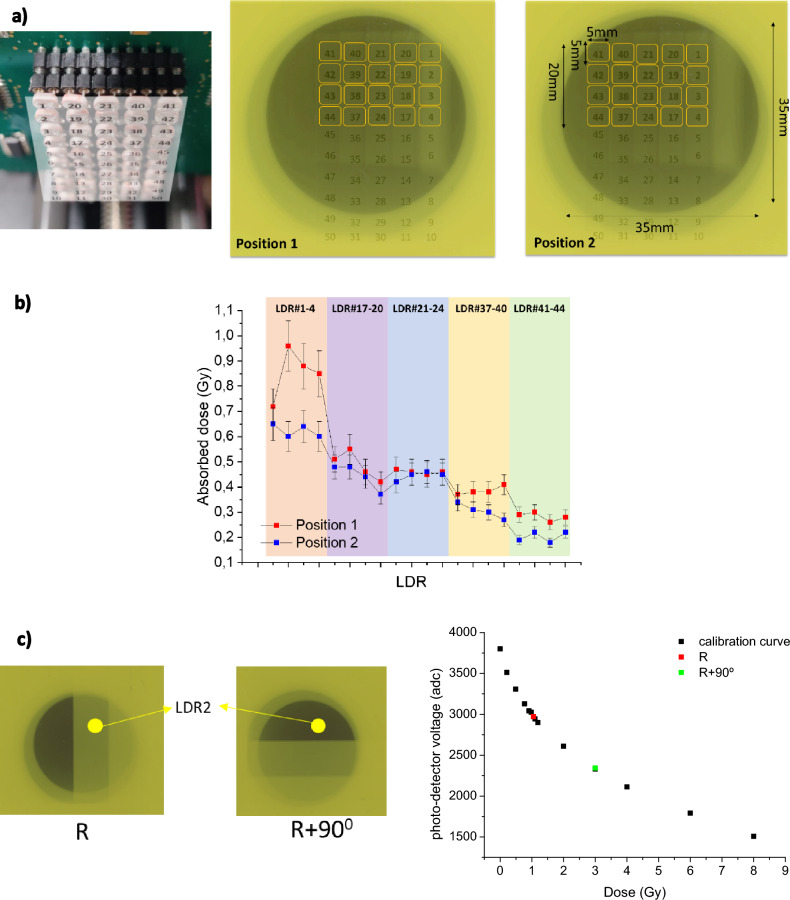
(**a**) schematic representation of each LDR and its position in the Gafchromic EBT3 film. Note that in the image of Gafchromic EBT3 film the LDRs are the specular image of the *dosiMOEMs*. (**b**) absorbed doses in each PMMA step obtained with *dosiMOEMs* for both positions. Each dot represents a #LDR number. (**c**) schematic representation for the position of LDR#2 in the Gafchromic EBT3 film for the two different orientations *R* and *R* + *90,* and graphic with the calibration curve obtained for LDR#2 with the dose obtained with the two orientations.

*DosiMOEMs* was able to distinguish heterogeneous dose distributions with the current resolution given by the LDR diameter, i.e. 5 mm (Fig. 5). Like in the case above, a proper analysis of the results requires the subtraction of the OD values with those obtained with an Gafchromic EBT3 film irradiated with the same dose (without the PMMA stairs). Fig. 5b shows the *dose-steps* with the PMMA in two different positions (Fig. 5a), shifted 5 mm between them. The LDRs placed in the same step (i.e., LDR#1-4, LDR#17-20, LDR#21-24, LDR#37-40, and LDR#41-44) should provide similar doses. Likewise, we obtain the same absorbed doses in *Position#2* (less the 5 mm shifted) than in *Position#1*. For instance, the LDR#41-44 at the *Position#1* recorded the same dose values than the LDR#37-40 at the *Position#2*. It is worth noting that when we displace the film from *Position#1* to *Position#2*, the doses recorded change slightly due to the uncertainties introduced by the manual alignment and the air gap between LDRs. Nevertheless, there is a disagreement in the LDR#1 value placed at the *Position#1:* it provided a different value that those recorded by its adjacent LDRs (#2, 3 and 4), since it is on the boundary of the irradiation zone and thus integrating lower doses and higher dose gradient (Fig. 5a). There are two LDR, namely #19 and #20, that have more error associated than the other LDRs. It is likely due to electronic issues of the corresponding ADC that are being investigated.

Table 2 summarizes the average doses deposited in the films in both positions. There is a very good agreement between the dose values obtained with both scanner and *dosiMOEMs* methods into the error bars. Moreover, was performed a Monte Carlo simulation and the dose ratios were compared with which obtained with *dosiMOEMs* (considering as reference the dose without PMMA staircase, i.e. 3 Gy), obtaining very similar dose ratio, which are in good agreement into the error bars.

Additionally, *dosiMOEMS* can be advantageous when compared to the scanner since it is able to distinguish *in-situ* the film orientation thanks to the automatized data analysis. To this end, the LDR#2 response was studied after irradiation through the Al staircase positioned at transversal orientations (Fig. 5c). When EBT3 film was orientated in the *R* position, the LDR#2 was in the (1.05 ± 0.1) Gy zone, which was in good agreement with the dose obtained with scanner (0.98 ± 0.1) Gy. Likewise, when EBT3 was orientated 90 degrees to the right, i.e. *R* + *90 position*, the LDR#2 fell into the 3 Gy zone, which was immediately detected by the *dosiMOEMs*.

### ^137^Cs irradiations

**Figure 6:**
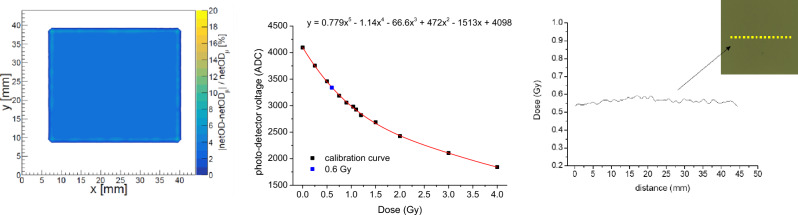
*Left* Homogeneity quantification of four representative EBT3 irradiated films at 4 Gy. *Medium* calibration curve using the *dosiMOEMs* (black dots) and ADC value related to a dose of 0.6 Gy (blue dot). *Right* Absorbed dose obtained with the scanner method once the corresponding calibration curve is applied. The average value is (0.56 ± 0.06) Gy along the irradiated area. The inset shows gafchromic films irradiated with the ^137^Cs source. The yellow dash lines represent the lateral sections where the dose profiles were extracted from.

In order to crosscheck the *dosiMOEMS* performance with another radiator with a homogeneous irradiation field, additional measurements were carried with a ^137^Cs source (“^137^Cesium source irradiations” section). Homogeneity of the ^137^Cs is depicted in Fig. 6, left. It is observed a dose variation lower than 4% along the irradiated surface, compared with the 20% showed in the X-ray radiator above.

Irradiated films herein were also calibrated with both *scanner* and *dosiMOEMs*. We obtained two different calibration curves with each method, which were extracted by fitting the data with a fifth-grade polynomic function, as detailed previously. In the case of *dosiMOEMs* (Fig. 6, medium), we normalized the 20 LDRs into only one, LDR#1.

As it was explained above, the use of both systems let us to determine unknown doses. Figure 6 shows a representative film irradiated at 0.6 Gy according to the ^137^Cs supplier calibration (Fig. 6, right, inset) and the corresponding absorbed dose obtained with the scanner (Fig. 6, right) and the *dosiMOEMs* (Fig. 6, medium, blue dot). Due to the homogeneity of the irradiated source and low percentage of OD dispersion, both the scanner and the *dosiMOEMs* provided very similar and accurate values after calibration (0.56 ± 0.06) and (0.6 ± 0.1) Gy for the scanner and the *dosiMOEMs*, respectively, confirming the reliability and robustness of the *dosiMOEMs* system in radiation dosage evaluation using gafchromic EBT3 films.

## Discussion

Radiochromic films do not allow real-time dosimetry, but they are one of the gold standards in QA in radiotherapy in a wide dose range due to their easy to handling and self-developing coloration. Regarding the daily QA requirements in the hospitals in radiotherapy, it might be useful to create new sensors that enable dose maps for fast readings. MOEMS have been widely used to design different types of biosensors^22^, but we are presenting the first one for radiotherapy. The feasibility of a new Micro-Opto-Electro-Mechanical system (MOEMs) based on a 5 × 10 sensor matrix for accurate dosimetry has been investigated. We found that the photo-resistive voltage of the MOEMS matrix works as a feasible physical parameter that allows us to quantify directly doses.

As it is shown in Table 2, there is a negligible difference in the dose values obtained from *dosiMOEMs* comparing with those obtained with a standard method (flatbed *scanner*). *DosiMOEMs* may distinguish the decreasing gradient of the dose due to the PMMA steps and evaluate the difference between same steps. The maximum dose difference between both methods is < 0.25 Gy, which is below the standard deviations. It means that our system may be used to determine the dose with the same accuracy than the flatbed *scanner*, but with the advantage of avoiding the elaborate process of acquisition, image processing and analyses used in the standard method. Moreover, dose values obtained were compared with Monte Carlo simulations, obtaining very good agreements.

In our first prototype the dose uncertainty reached 7%, higher than with the standard method 3‒7%^13^. Several improvements have been performed to reduce the overall uncertainty of the system up to an intrinsic resolution (LED + LDR + ADC) lower than 0.2%. Consequently, the opto‒electro parameter delivered by the MOEMS could be used for a faster processing of dose evaluation instead of the optical density.

There are still design issues to be figured out, for example: (i) the current prototype has a poor resolution (5 mm) due to the size limitations of the commercial photoresistors (> 5 mm diameter) and (ii) the doses recorded may change slightly due to the manual alignment and the air gap between LDRs. It is being corrected by designing and manufacturing *ad-hoc* photoresistors (< 1 mm) in the IMB-CNM clean-room facility to be integrated into the electronics and including a mechanical frame to fix the film positions. Moreover, we are developing a customized readout electronics to create an advanced prototype (16 × 16 LDRs) to cover up to 1.6 cm × 1.6 cm radiation sensitive area. In addition, we plan to integrate it in a flexible printed circuit to be placed laterally and thus avoiding interferences between the radiation and the radiochromic film. At the same time, we are developing an extended in‒house graphical user interface, python based, for the post‒processing of the matrix outcomes in real-time. Additionally, as the optical density of the Gafchromic EBT3 films changes over time after irradiation, the MOEMS response to this variation would imply an unavoidable temporal delay. This is a common limitation of the current readout methods for the radiochromic films analysis. We are facing this issue by developing a novel kind of radiochromic films whose colour does not change with the time. This, added to the extended *dosiMOEMs* configuration above, would allow us to perform dose verifications in real-time and in-situ in clinical scenarios.

To the best of our knowledge, there are only few alternatives of dose verification based on gafchromic films in real-time^23–27^. For instance, Casolaro et al. proposed a set-up based on optical fibres^25^, where the films are located on the tip of fibres and the light is backscattered after passing into the films and the sent to a spectrometer. The last study of this group has demonstrated the radiation hard feature of the system (from a few Gy to a few MGy)^26^. Nevertheless, the features of this system make difficult its use for daily dose monitoring due to the bulky instruments used (spectrometer and power light source) and particularly since it is able to measure only one spot. On the other hand, Yasuda et al.^27^, have recently proposed a method for reading radiochromic films by using a portable colorimeter that has been tested in X-rays. They obtained a low variability (< 3%) although with some angle dependence, but they are also limited to one-spot measurements. Neither of them enables two-dimensional dose measurements. In contrast, thanks to the high electronic versatility of the MOEM system, we may extend the modulable configuration to reach multiple sensitive spots, i.e. having high spatial resolution and covering large radiation areas.

## Conclusions

We have created a MOEM based system that is able to deliver dose distributions in two-dimensions without post-processing. *DosiMOEM*s can quantify the same dose gradients of the EBT3 irradiated films than the standard method. Therefore, it can be used as a practical on-site dosimetry. The modulable MOEM configuration is paving the way to perform novel real-time dosimeters in radiotherapy.

Since radiochromic films are widely used not only in beam diagnostics, but also in industry and research, e.g. in radiation-induced sterilization, studies of electronics radiation damage, etc., our devices can also be too employed in these fields.

## Data Availability

The data analysed during the current study are available from the corresponding author.
